# Prognostic Impact of Different Gleason Patterns on Biopsy Within Grade Group 4 Prostate Cancer

**DOI:** 10.1245/s10434-021-10257-x

**Published:** 2021-06-11

**Authors:** Keiichiro Mori, Vidit Sharma, Eva M. Comperat, Shun Sato, Ekaterina Laukhtina, Victor M. Schuettfort, Benjamin Pradere, Reza Sari Motlagh, Hadi Mostafaei, Fahad Quhal, Mehdi Kardoust Parizi, Mohammad Abufaraj, Pierre I. Karakiewicz, Shin Egawa, Derya Tilki, Stephen A. Boorjian, Shahrokh F. Shariat

**Affiliations:** 1 Department of Urology, Medical University of Vienna, Vienna, Austria; 2 Department of Urology, The Jikei University School of Medicine, Tokyo, Japan; 3 Department of Urology, Mayo Clinic, Rochester, MN USA; 4 Department of Urology, VA Health Services Research and Development Fellowship, University of California, Los Angeles, CA USA; 5 Department of Pathology, Hôpital Tenon, Sorbonne University, Paris, France; 6 Department of Pathology, The Jikei University School of Medicine, Tokyo, Japan; 7 Institute for Urology and Reproductive Health, Sechenov University, Moscow, Russia; 8 Department of Urology, University Medical Center Hamburg-Eppendorf, Hamburg, Germany; 9 Department of Urology, University Hospital of Tours, Tours, France; 10 Men’s Health and Reproductive Health Research Center, Shahid Beheshti University of Medical Sciences, Tehran, Iran; 11 Research Center for Evidence-Based Medicine, Tabriz University of Medical Sciences, Tabriz, Iran; 12 Department of Urology, King Fahad Specialist Hospital, Dammam, Saudi Arabia; 13 Department of Urology, Shariati Hospital, Tehran University of Medical Sciences, Tehran, Iran; 14 Division of Urology, Department of Special Surgery, The University of Jordan, Amman, Jordan; 15 Cancer Prognostics and Health Outcomes Unit, University of Montreal Health Centre, Montreal, QC Canada; 16 Martini-Klinik Prostate Cancer Center, University Hospital Hamburg-Eppendorf, Hamburg, Germany; 17 Department of Urology, University Hospital Hamburg-Eppendorf, Hamburg, Germany; 18 Department of Urology, Weill Cornell Medical College, New York, NY USA; 19 Department of Urology, University of Texas Southwestern, Dallas, TX USA; 20 Karl Landsteiner Institute of Urology and Andrology, Vienna, Austria; 21 Department of Urology, Second Faculty of Medicine, Charles University, Prague, Czech Republic; 22 European Association of Urology Research Foundation, Arnhem, The Netherlands

## Abstract

**Background:**

Grade group (GG) 4 prostate cancer (PC) is considered a single entity; however, there are questions regarding prognostic heterogeneity. This study assessed the prognostic differences among various Gleason scores (GSs) classified as GG 4 PC on biopsy before radical prostatectomy (RP).

**Methods:**

We conducted a multicenter retrospective study, and a total of 1791 patients (GS 3 + 5: 190; GS 4 + 4: 1557; and GS 5 + 3: 44) with biopsy GG 4 were included for analysis. Biochemical recurrence (BCR)-free survival, cancer-specific survival, and overall survival were analyzed using the Kaplan–Meier method and the log-rank test. Logistic regression analysis was performed to identify factors associated with high-risk surgical pathologic features. Cox regression models were used to analyze time-dependent oncologic endpoints.

**Results:**

Over a median follow-up of 75 months, 750 patients (41.9%) experienced BCR, 146 (8.2%) died of any causes, and 57 (3.2%) died of PC. Biopsy GS 5 + 3 was associated with significantly higher rates of GS upgrading in RP specimens than GS 3 + 5 and GS 4 + 4. On multivariable analysis adjusted for clinicopathologic features, different GSs within GG 4 were significantly associated with BCR (*p *= 0.03) but not PC-specific or all-cause mortality. Study limitations include the lack of central pathological specimen evaluation.

**Conclusions:**

Patients with GG 4 at biopsy exhibited some limited biological and clinical heterogeneity. Specifically, GS 5 + 3 had an increased risk of GS upgrading. This can help individualize patients’ counseling and encourage further study to refine biopsy specimen-based GG classification.

**Supplementary Information:**

The online version contains supplementary material available at 10.1245/s10434-021-10257-x.

Several studies have shown that a higher Gleason score (GS) is an important prognostic factor for prostate cancer (PC) regardless of treatment.^1^–^3^ Tumor grading was reported using Grade Groups (GGs) first proposed by authors at Johns Hopkins Hospital,^2^ validated in a large multi-institutional study,^4^ and subsequently endorsed by the 2014 International Society of Urological Pathology (ISUP) Consensus Conference,^5^ whereby GG1 = GS ≤ 6, GG2 = GS 3 + 4 = 7, GG3 = GS 4 + 3 = 7, GG4 = GS 8, and GG5 = GS 9–10. The GGs reflect a biological and clinical behavioral distinction within PCs with GS 7, differentiating between GS 3 + 4 (GG 2) and GS 4 + 3 (GG 3).^6^

Currently, GG 4 is equivalent to GS 8, consisting of GSs 4 + 4, 3 + 5, and 5 + 3. GG 4 is still considered a homogenous entity with regard to its associated prognosis and treatment allocation. However, some reports have raised questions regarding its prognostic heterogeneity, suggesting the reclassification of GG 4 into separate GGs.^7^,^8^ Moreover, given that Gleason pattern 5 has a negative prognostic significance compared with pattern 4, there is concern that GG 4 is subject to heterogeneity with respect to oncological outcomes.^9^–^11^ An updated meta-analysis of different GS patterns of PC in GG 4 showed that GS 4 + 4 was associated with better overall survival (OS);^12^ however, this meta-analysis had significant heterogeneity in the population of interest, mainly because it did not restrict the interventions implemented. Moreover, it made no distinction between GG 4 on biopsy and radical prostatectomy (RP) specimens.

We have shown prognostic differences in patients with PC within GG 4 treated with RP based on different GSs in RP specimens, suggesting that there is considerable heterogeneity within GG 4 in terms of oncological and surgical pathologic outcomes.^13^ However, there is large discrepancy between the biopsy and RP GS, with the two specimens matching exactly in only approximately 40–60% of cases.^14^–^17^ Thus, it remains unclear whether our findings on RP specimens also hold true for biopsy specimens.

As treatment decisions are generally made based on prostatic biopsy specimens, adequate biopsy GS stratification remains of utmost importance. Therefore, the purpose of this study was to determine the prognostic homogeneity/heterogeneity, as assessed by pathologic and oncologic outcomes, between the three different GS groups within biopsy GG 4 in patients treated with RP. Such analyses may enable more accurate risk stratification of patients with biopsy GG 4.

## Material and Methods

### Patient Selection

This study obtained approval from the Institutional Review Board at each participating institution, with all sites providing institutional data-sharing agreements prior to the initiation of the study. A total of 6724 patients were treated with RP for clinically nonmetastatic PC between 2005 and 2019 at the four participating institutions (Mayo Clinic, University Hospital Hamburg-Eppendorf, Weill Cornell Medical College, and University of Texas Southwestern). Patients with biopsy GG 4 (consisting of GSs 4 + 4, 3 + 5, and 5 + 3) were then included for analysis; as such, a total of 1791 patients were assessed. No patients received neoadjuvant hormone therapy. The multicenter retrospective nature of the study meant that preoperative staging was not standardized. In general, preoperative imaging (conventional bone scans, computed tomography [CT] scan of the chest, abdomen, and pelvis) was performed based on the patients’ clinicopathological features (i.e. prostate-specific antigen [PSA] and GS at biopsy), current guidelines, and physician discretion. Patients were considered to have non-metastatic disease if preoperative imaging showed no cancer spread from the primary site to other sites.

### Data Collection and Pathologic Evaluation

Demographic, surgical, pathologic, and outcome data were collected. Data on age, biopsy GS, clinical stage, baseline PSA, RP GS, pathologic stage, and positive surgical margins (PSMs) were confirmed for all patients. Specimens were analyzed by dedicated genitourinary pathologists at each center. Pathologic stage was assigned using the 2009 American Joint Committee on Cancer tumor-node-metastasis staging system.

### Management and Follow-Up

All patients were treated with RP with or without pelvic lymph node dissection according to guideline recommendations at the time of the study and at the surgeons’ discretion. While the multicenter retrospective nature of the study meant that the area of lymph node dissection was not standardized, as a rule, extended lymph node dissection was performed in the current cohort, which included only high-risk PC. Patients were followed-up in accordance with institutional protocols and local guidelines at the time. In general, patients underwent physical examinations and PSA testing every 3 months in the first year after surgery, semi-annually from the second to fifth years, and annually thereafter. Biochemical recurrence (BCR) was defined as two consecutive increases in PSA over 0.2 ng/mL.^18^ The date of the first increase was considered the date of BCR. The cause of death was determined by the treating physician, based on chart reviews corroborated by death certificates, or by death certificates alone. Follow-up time was calculated as starting from the date of RP.

### Statistical Analysis

Associations of GS with categorical variables were assessed using the Chi-square test or Fisher’s exact test, and differences in continuous variables were analyzed using the Kruskal–Wallis test. BCR-free survival (BCRFS), cancer-specific survival (CSS), and OS were analyzed using the Kaplan–Meier method and the log-rank test. Extraprostatic extension (EPE) was defined as ≥ *p*T3a, while non-organ-confined (NOC) disease was defined as  ≥ *p*T3a and/or lymph node-positive disease. Logistic regression analysis was performed to assess the association of GS and other predictive factors with GS upgrading, PSM, lymph node metastasis, EPE, and NOC disease. Univariable and multivariable Cox regression models were used to evaluate the association of various prognostic factors with BCR, death from PC, and all-cause mortality. The discrimination of the model was evaluated using Harrel’s concordance index. All *p*-values were two-sided and statistical significance was defined as *p *< 0.05. Statistical analyses were performed using R (The R Foundation for Statistical Computing, Vienna, Austria) and Stata/MP 14.3 statistical software (StataCorp LLC, College Station, TX, USA).

## Results

### Patient Demographics and their Association with the Gleason Score (GS)

A total of 1791 patients (GS 3 + 5, 190; GS 4 + 4, 1557; and GS 5 + 3, 44) were included in the analysis. Table 1 and electronic supplementary Table 1 summarize the clinicopathological characteristics of the study cohort. Lymphadenectomy was performed in 1773 patients (99.0%). There was a significant difference in RP GS and pathological node stage between the groups (*p *< 0.001 and *p* = 0.02, respectively). Biopsy GS 5 + 3 was associated with higher rates of GS upgrading and lower rates of GS downgrading in RP specimens than GS 4 + 4 and GS 3 + 5 (*p* = 0.0009) [Table 2]. Electronic supplementary Table 2 summarizes the clinicopathologic characteristics of the patients at each institution. Heterogeneity was found in patient characteristics between the participating institutions, with that in the proportion of patients receiving adjuvant treatments (androgen deprivation therapy [ADT] 0–14.6%; radiation therapy [RT] 0–9.1%) that could have affected survival and in the proportion of patients with GS 3 + 5 (6.4–17.6%), GS 4 + 4 (78.4–92.4%), and GS 5 + 3 (1.2–4.1%) in GG 4 found to be particularly large.

**Table 1:** Patient demographics

	All	Biopsy GS 3 + 5	Biopsy GS 4 + 4	Biopsy GS 5 + 3	*p*-value
Number of patients	1791	190	1557	44	
Median age, years (IQR)	66 (61–70)	65.5 (60–69)	66 (61–70)	64 (58–68.5)	0.11
Median preoperative PSA (IQR)	7.8 (5.3–12.3)	7.0 (5–11.1)	7.9 (5.4–12.3)	8.1 (5.3–18.3)	0.09
cT stage					0.64
cT1	1010 (56.4)	118 (62.1)	868 (55.7%)	24 (54.5%)	
cT2	700 (39.1)	66 (34.7%)	617 (39.6%)	17 (38.6%)	
≥ cT3	44 (2.5)	6 (3.2%)	37 (2.4%)	1 (2.3%)	
Missing	37 (2.1)	0	35 (2.2%)	2 (4.5	
Median number of biopsy cores (IQR)	10 (6–12)	10 (5–12)	10 (6–12)	10 (6.5–11)	0.44
Median number of positive biopsy cores (IQR)	4 (1–6)	4 (1–6)	4 (1–6)	4 (3–7)	0.37
Median extent of core involvement (IQR)	60 (30–90)	80 (60–95)	60 (30–87.5)	60 (45–75)	**0.02**
RP GS					**<0.001**
GG1	18 (1.0)	3 (1.6)	14 (0.9)	1 (2.3)	
GG2	489 (27.3)	75 (39.5)	403 (25.9)	11 (25.0)	
GG3	711 (39.7)	51 (26.8)	654 (42.0)	6 (13.6)	
GG4	244 (30.8)	28 (14.7)	209 (13.4)	7 (15.9)	
GG5	322 (18.0)	33 (17.4)	271 (17.4)	18 (40.9)	
Missing	7 (0.4)	0	6 (0.4)	1 (2.3)	
*p*T stage					0.20
≤ *p*T2	783 (43.7)	80 (42.1)	689 (44.3)	14 (31.8)	
*p*T3a	629 (35.1)	75 (39.5)	539 (34.6)	15 (34.1)	
≥ *p*T3b	373 (20.8)	35 (18.4)	324 (20.8)	14 (31.8)	
Missing	6 (0.3)	0	5 (0.3)	1 (2.3)	
*p*N stage					**0.02**
*N*_0_	1443 (80.6)	155 (81.6)	1260 (80.9)	28 (63.6)	
*N*_1_	330 (18.4)	33 (17.4)	282 (18.1)	15 (34.1)	
*Nx*	18 (1.0)	2 (1.1)	15 (1.0)	1 (2.3)	
PSM	447 (25.0)	49 (25.8)	384 (24.7)	14 (31.8)	0.54
Adjuvant ADT	113 (6.3)	13 (6.8)	92 (5.9)	8 (18.2)	**0.004**
Adjuvant RT	122 (6.8)	20 (10.5)	99 (6.4)	3 (6.8)	0.10
Median follow-up, months (IQR)	75 (48–101)	86 (50.3–11.5)	75 (47.2–100)	88 (53.4–109.8)	

Data are expressed as *n* (%) unless otherwise specified

*ADT* androgen deprivation therapy, *cT stage* clinical T stage, *GS* Gleason score, *IQR* interquartile range, *pT stage* pathological T stage, *PSA* prostate-specific antigen, *PSM* positive surgical margin, *RP* radical prostatectomy, *RT* radiation therapy

Bold *p* values are considered statistically significant (*p* value < 0.05)

**Table 2:** Concordance between biopsy and RP specimens

	Biopsy			*p*-value
	GS 3 + 5	GS 4 + 4	GS 5 + 3	**0.0009**
*RP*				
Downgrading GS GG 1, 2, or 3)	129 (67.9)	1071 (69.1)	18 (41.9)	
Same GS (GG 4)	28 (14.7)	209 (13.5)	7 (16.3)	
Upgrading GS (GG 5)	33 (17.4)	271 (17.5)	18 (41.9)	

Data are expressed as *n* (%)

*GG* grade group, *GS* Gleason score, *RP* radical prostatectomy

Bold *p* values are considered statistically significant (*p* value < 0.05)

### Association between the GS and High-Risk Surgical Pathological Features

On multivariable analyses adjusting for PSA and clinical T stage, biopsy GS within GG 4 was significantly associated with GS upgrading in RP specimens (*p* = 0.004), but not with the risks of PSM, lymph node metastasis, EPE, and NOC disease (Table 3). Specifically, compared with GS 3 + 5, GS 5 + 3 was significantly associated with higher rates of GS upgrading in RP specimens (odds ratio [OR] 3.24, 95% confidence interval (CI) 1.54–6.83; *p* = 0.002). Similarly, biopsy GS 5 + 3 was significantly associated with higher rates of GS upgrading in RP specimens than GS 4 + 4 (OR 3.17, 95% CI 1.65–6.08; *p* = 0.0005).

**Table 3:** Logistic regression analysis (adjusting PSA and clinical T stage)

	Univariable analysis		Multivariable analysis	
	OR (95% CI)	*p*-value	OR (95%CI)	*p*-value
*RP GS upgrading*				
GG 4 Reference GS 3 + 5		**0.0012**		**0.004**
GS 4 + 4	1.01 (0.68–1.50)	0.97	0.92 (0.68–1.54)	0.92
GS 5 + 3	3.43 (1.68–6.99)	**0.0007**	3.24 (1.54–6.83)	**0.002**
Reference GS 4 + 4				
GS 5 + 3	3.40 (1.83–6.32)	**0.0001**	3.17 (1.65–6.08)	**0.0005**
*Extraprostatic extension*				
GG 4 Reference GS 3 + 5		0.26		0.33
GS 4 + 4	0.91 (0.67–1.24)	0.55	0.83 (0.60–1.15)	0.27
GS 5 + 3	1.51 (0.75–3.03)	0.25	1.21 (0.57–2.58)	0.63
Reference GS 4 + 4				
GS 5 + 3	1.65 (0.87–3.15)	0.13	1.45 (0.72–2.93)	0.30
*Lymph node metastasis*				
GG 4 Reference GS 3 + 5		**0.04**		0.18
GS 4 + 4	1.05 (0.71–1.56)	0.81	1.03 (0.67–1.58)	0.89
GS 5 + 3	2.52 (1.21–5.23)	**0.01**	2.10 (0.92–4.76)	0.08
Reference GS 4 + 4				
GS 5 + 3	2.39 (1.26–4.54)	**0.008**	2.03 (0.98–4.20)	0.06
*Non-organ confined disease*				
GG 4 Reference GS 3 + 5		0.21		0.28
GS 4 + 4	0.92 (0.68–1.25)	0.61	0.83 (0.60–1.16)	0.28
GS 5 + 3	1.60 (0.79–3.28)	0.19	1.30 (0.60–2.81)	0.50
Reference GS 4 + 4				
GS 5 + 3	1.74 (0.90–3.37)	0.10	1.56 (0.76–3.19)	0.22
*Positive surgical margin*				
GG 4 Reference GS 3 + 5		0.53		0.82
GS 4 + 4	0.94 (0.66–1.32)	0.71	0.90 (0.63–1.31)	0.59
GS 5 + 3	1.35 (0.66–2.76)	0.41	1.03 (0.46–2.30)	0.94
Reference GS 4 + 4				
GS 5 + 3	1.44 (0.75–2.75)	0.27	1.14 (0.55–2.37)	0.72

*CI* confidence interval, *GG* grade group, *GS* Gleason score, *OR* odds ratio, *PSA* prostate-specific antigen, *RP* radical prostatectomy

Bold *p* values are considered statistically significant (*p* value < 0.05)

### Association between the GS, Recurrence, and Survival

**Fig. 1:**
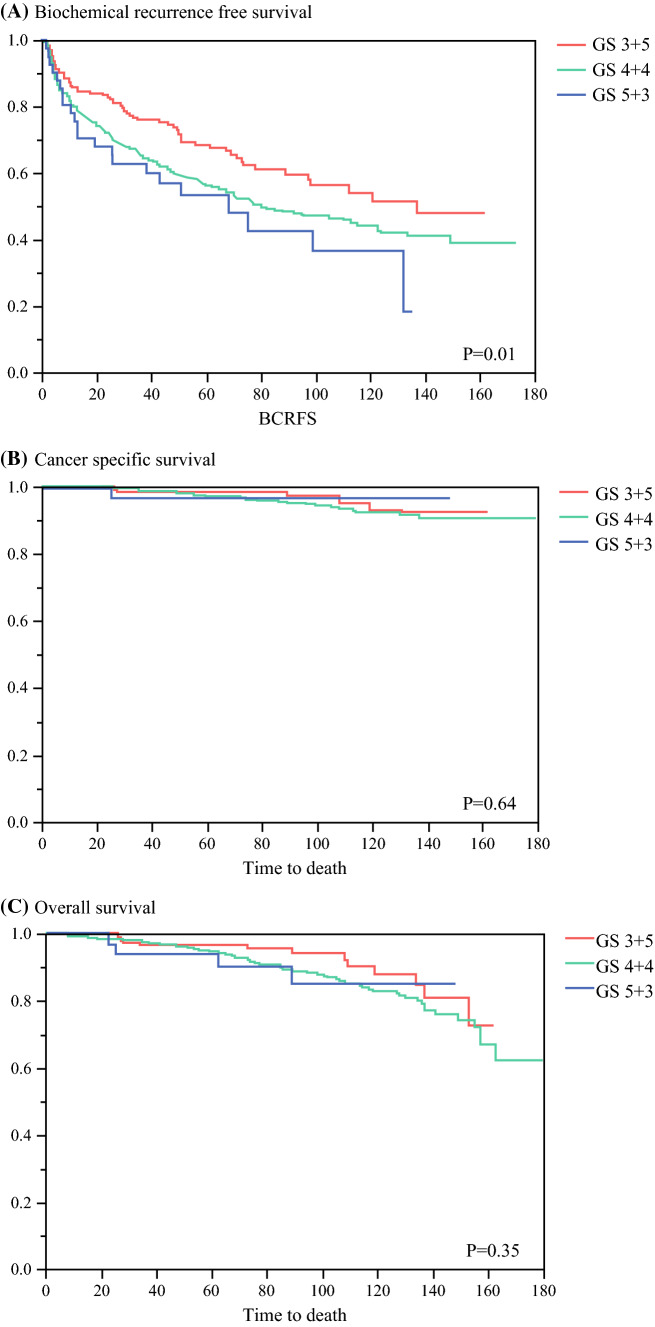
Kaplan–Meier estimates of oncologic outcomes stratified by different Gleason scores in 1791 prostate cancer patients with grade group 4 treated with radical prostatectomy. **a** Biochemical recurrence free survival; **b** overall survival; **c** cancer-specific survival. *GS* Gleason scores, *BCRFS* biochemical recurrence-free survival

At a median follow-up of 75 months, 750 patients experienced BCR, 146 died of any cause, and 57 died of PC. GS was significantly associated with BCRFS in the log-rank analysis (*p* = 0.01) [Fig. 1]. The BCRFS rates in biopsy GS 3 + 5, 4 + 4, and 5 + 3 were 61.2%, 49.0%, and 42.7%, respectively, at the 7-year follow-up, and 51.5%, 44.2%, and 36.6%, respectively, at the 10-year follow-up. Table 4 shows the results of the univariable and multivariable Cox proportional hazard regression analyses in the overall cohort. In the univariable analysis, GS was significantly associated with BCRFS (*p *= 0.007). In the multivariable analysis that adjusted for clinicopathologic features, GS remained an independent prognostic factor for BCRFS (*p *=  0.03). In contrast, GS was not associated with OS and CSS. Compared with GS 3 + 5, GS 4 + 4 was significantly associated with worse BCRFS (hazard ratio 1.43, 95% CI 1.12–1.86: *p *= 0.005). Adding the GS did not improve the accuracy of the predictive models for BCRFS, OS, or CSS (data not shown).

**Table 4:** Cox regression analysis (adjusting PSA and pathological T stage and PSM)

	Univariable analysis		Multivariable analysis	
	HR (95% CI)	*p*-Value	HR (95%CI)	*p*-value
*Biochemical recurrence-free survival*				
GG 4 Reference GS 3 + 5		**0.007**		**0.03**
GS 4 + 4	1.44 (1.13–1.88)	**0.003**	1.43 (1.12–1.86)	**0.005**
GS 5 + 3	1.78 (1.07–2.84)	**0.03**	1.38 (0.83–2.20)	0.21
Reference GS 4 + 4				
GS 5 + 3	1.23 (0.78–1.84)	0.35	0.97 (0.61–1.45)	0.89
*Overall survival*				
GG 4 Reference GS 3 + 5		0.32		0.25
GS 4 + 4	1.52 (0.89–2.82)	0.13	1.65 (0.95–3.16)	0.08
GS 5 + 3	1.56 (0.44–4.43)	0.45	1.49 (0.42–4.31)	0.50
Reference GS 4 + 4				
GS 5 + 3	1.03 (0.32–2.45)	0.95	0.91 (0.28–2.16)	0.84
*Cancer-specific survival*				
GG 4 Reference GS 3 + 5		0.61		0.53
GS 4 + 4	1.48 (0.65–4.27)	0.38	1.51 (0.66–4.35)	0.36
GS 5 + 3	0.93 (0.05–5.75)	0.94	0.71 (0.04–4.44)	0.75
Reference GS 4 + 4				
GS 5 + 3	0.63 (0.04–2.85)	0.62	0.47 (0.03–2.16)	0.40

*CI* confidence interval, *GG* grade group, *GS* Gleason score, *HR* hazard ratio, *PSA* prostate-specific antigen, *PSM* positive surgical margin

Bold *p* values are considered statistically significant (*p* value < 0.05)

## Discussion

This study was conducted to investigate the prognostic differences between GS 3 + 5, GS 4 + 4, and GS 5 + 3 in biopsy specimens from patients with PC classified into GG 4 based on the association with oncologic and surgical pathologic outcomes. The results indicate that GS 5 + 3 was associated with significantly higher rates of GS upgrading in RP specimens than GS 3 + 5 and GS 4 + 4. In contrast, GS was not associated with lymph node metastases, NOC, PSM, and EPE disease. Moreover, GS was not associated with OS or CSS, but was significantly associated with BCRFS.

Initial validation studies of grading for PC combined GS 8 into one prognostic group;^5^ however, the results from our study do not provide clear support for subdividing patients with GS 8 into three prognostic groups. Current evidence suggests that, as the strongest pathologic predictor of recurrence, metastasis, and PC-specific death, Gleason pattern 5 may have important biological and clinical implications and accounts for varying oncological outcomes in patients who fall within the GG 4 category.^9^,^10^,^19^ In addition, GS 3 + 4 and GS 4 + 3 patterns differ significantly in prognosis depending on the percentage of Gleason pattern 4 cancer present (greater or less than 50%), suggesting that the percentage of Gleason pattern 5 cancer may result in differences in prognosis between the GS 3 + 5 and GS 5 + 3 patterns, in agreement with previous studies demonstrating that the percentage of high-grade patterns has prognostic value in predicting oncological outcomes in PC patients undergoing RP.^20^–^22^ Therefore, the proposal to classify patients with GS 8 into a single category (GG 4) may not have strong theoretical support. However, our biopsy specimen-based study detected some limited differences within the GG 4 category in terms of BCR and GS upgrading in RP specimens.

Our findings are relevant considering the paucity of studies assessing the prognostic differences within GG 4 in patients with PC treated with RP.^7^,^13^,^23^–^25^ Indeed, a review of the literature shows that the evaluation of GG 4 involved biopsy specimens alone in one study, prostatectomy specimens alone in two studies, and biopsy/prostatectomy specimens in two studies (electronic supplementary Table 3); however, of these, the study involving biopsy specimens alone evaluated GS downgrading as the only outcome measure, but provided no survival analysis.^24^ Therefore, this is the first analysis to assess differences in prognosis in terms of mortality, BCR, and surgical pathological outcomes among PC patients within the GG 4 category (GS 3 + 5 vs. GS 4 + 4 vs. GS 5 + 3) treated with RP based on biopsy specimens. In this regard, the limited heterogeneity shown in this study within GG 4 in terms of oncological and surgical pathological outcomes have clinically relevant implications in patients who fall within the GG 4 category.

Again, while the RP specimen-based studies reported not only oncological but also surgical pathologic outcomes, biopsy GS remains the mainstay of diagnosis as a basis for treatment decision making. In addition, there is large discrepancy between biopsy and RP GS, with the concordance rate between these GSs reported to be no more than 40–60%.^14^–^17^ Moreover, GS is differently assigned in an RP specimen than in a biopsy specimen due to the much larger area of tissue sampled and the different pathological criteria used for grade assignment in biopsy and RP specimens. For example, GS is differently assigned in patients whose secondary Gleason pattern is assigned a higher GS despite accounting for < 5% of the tumor, or in patients whose tertiary Gleason pattern is GS 5 despite accounting for < 5% of the tumor. Indeed, the current study showed different results from those of our previous RP specimen-based study,^13^ suggesting that biopsy specimens are not sufficiently accurate to yield similar results relative to RP specimens, and thus leading to minimal heterogeneity between GS patterns within biopsy GG 4 (GS 3 + 5 vs. GS 4 + 4 vs. GS 5 + 3) despite their significant difference in regard to GS upgrading, downgrading, or BCR.

While this study provides a number of findings of interest, it has some limitations. First, the pathological specimens were not centrally evaluated, and most patients depended on their individual pathologists for GS identification and reporting. Furthermore, this retrospective study failed to evaluate the percentage of each Gleason grade in biopsy specimens, thus possibly affecting survival outcomes. Moreover, the GS patterns were shown to be differently distributed in our study than previously reported. In an earlier large multi-institutional study involving genitourinary pathologists and conducted from 2005 to 2014, of the 16,172 patients undergoing needle biopsies, only 44 (0.3%) and 6 (0.04%) were shown to have GS 3 + 5 = 8 and GS 5 + 3 = 8, respectively (unpublished data).^26^ In contrast, in our study, a majority (86.9%) of the patients had GS 4 + 4, while 10.6% and 2.5% had GS 3 + 5 and GS 5 + 3, respectively. Moreover, the study has found large inter-institutional heterogeneity in the proportion of patients shown to have GS 3 + 5 (range 6.4–17.6%), GS 4 + 4 (range 78.4–92.4%), and GS 5 + 3 (range 1.2–4.1%). Thus, the proportions of patients shown to have GS 3 + 5 and GS 5 + 3 varied from one institution to the next but were high at all institutions. This raises concern as to whether or not our findings may be readily generalizable. The absence of central reviews involving expert pathologists may thus be the largest limitation of this study, given that, indeed, earlier studies lacking central reviews were associated with high proportions of patients with GS 3 + 5 and GS 5 + 3 as our study (electronic supplementary Table 3), and that a high percentage of GS 3 + 5 and GS 5 + 3 has been re-categorized upon expert review.^27^ These factors could have led to the misinterpretation of the pathological reports, thus unpredictably affecting the oncologic outcomes. Second, the preoperative staging, operation method, and follow-up protocols could not be standardized. Moreover, due to its multicenter nature, our study may have suffered from heterogeneity in the selection of patients and administration of adjuvant and salvage treatments. Indeed, it was found to be particularly large in the proportion of patients undergoing adjuvant treatments (ADT 0–14.6%; RT 0–9.1%), which could have affected survival outcomes. Third, given its multi-institutional nature, many institutional characteristics, which likely remained only insufficiently captured by our regression models, may have affected the study outcomes (e.g. inherent differences in follow-up protocols, preoperative staging, operation method, monitoring of oncologic events, and pathologic specimen processing). Thus, overrepresentation of one of these institutions within one of the three subclassifications of GG 4 could have skewed the results. Fourth, some relevant data (e.g. MRI image data or use of targeted fusion biopsies) were unavailable for analysis. Moreover, lack of patient data on factors deemed important to pathological evaluation, such as cribriform or intraductal features, was also a major limitation of the study. Fifth, the inclusion of subjects with high PSA levels in the study cohort may have led to selection or information bias in this study. Furthermore, limitations associated with the use of conventional imaging modalities have been highlighted in this study. Finally, given the median follow-up duration of 75 months and the low number of deaths, this study may not have evaluated mortality adequately.

## Conclusion

We found that patients with biopsy GG 4 exhibited some limited heterogeneity, while significant differences were seen in association with GS upgrading, downgrading, or BCR. Therefore, the biopsy specimen-based GG 4 classification may be deemed valid. However, caution should be exercised in interpreting the conclusions drawn from this study, given the lack of central pathological specimen evaluation. Thus, well-designed prospective studies with prolonged follow-up are warranted to validate the differential prognostic and biological values within GG 4 in the clinical setting, as well as to investigate whether such validation may lead to better clinical decision making for patients with PC.

## Supplementary Information

Below is the link to the electronic supplementary material.

Supplementary file1 (DOCX 21 kb)

Supplementary file2 (DOCX 22 kb)

Supplementary file3 (DOCX 17 kb)

Supplementary file4 (DOCX 40 kb)
