# Supplementary material for: Prognostic Impact of Different Gleason Patterns on Biopsy Within Grade Group 4 Prostate Cancer
Source: Ann Surg Oncol. 2021 Jun 11;28(13):9179–87. doi: 10.1245/s10434-021-10257-x (PMC8591010; doi:10.1245/s10434-021-10257-x)
Supplement: Supplementary file 1 — Supplementary file1 (DOCX 21 kb) [file 10434_2021_10257_MOESM1_ESM.docx]

Supplementary Table 1

Additional patient demographics

|  | ALL | Biopsy GS 3+5 | Biopsy GS 4+4 | Biopsy GS 5+3 | *P* |
| --- | --- | --- | --- | --- | --- |
| Number | 1791 | 190 | 1557 | 44 |  |
| Race Caucasian | 1528 (85.3%) | 163 (85.8%) | 1328 (85.3%) | 37 (84.1%) | 0.45 |
| Black or  African American | 7 (0.4%) | 1 (0.5%) | 6 (0.4%) | 0 |  |
| Other | 10 (0.6%) | 2 (1.1%) | 7 (0.4%) | 1 (2.3%) |  |
| missing | 246 (13.7%) | 24 (12.6%) | 216 (13.9%) | 6 (13.6%) |  |
| Family history |  |  |  |  |  |
| Yes | 205 (11.4%) | 28 (14.7%) | 174 (11.2%) | 3 (6.8%) | 0.27 |
| No | 676 (37.7%) | 67 (35.3%) | 594 (38.2%) | 15 (34.1%) |  |
| missing | 910 (50.8%) | 95 (50.0%) | 789 (50.7%) | 26 (59.1%) |  |
| Median BMI (IQR) | 27.0 (24.8-29.9) | 26.4 (24.7-29.0) | 27  (24.8-30.1) | 26.0  (24.8-28.4) | 0.19 |
| Active Surveillance |  |  |  |  |  |
| Yes | 22 (1.2%) | 3 (1.6%) | 19 (1.2%) | 0 | 0.74 |
| No | 461 (25.7%) | 51 (26.8%) | 400 (25.7%) | 10 (22.7%) |  |
| missing | 1308 (73.0%) | 136 (71.6%) | 1138 (73.1%) | 34 (77.3%) |  |
| Median P-Volume (IQR) | 40 (30-51.45) | 38 (30-48.5) | 40 (30-52) | 39.5 (31.3-55.8) | 0.50 |
| Operation method |  |  |  |  |  |
| Open | 1334 (74.5%) | 137 (72.1%) | 1162 (74.6%) | 35 (79.5%) | 0.81 |
| Laparoscopic | 249 (13.9%) | 28 (14.7%) | 217 (13.9%) | 4 (9.1%) |  |
| Robotic | 208 (11.6%) | 25 (13.2%) | 178 (11.4%) | 5 (11.4%) |  |
| Median number of  removed LN (IQR) | 13 (7-21) | 14 (7-21) | 13 (7-20.8) | 13 (10-21) | 0.97 |
| Abbreviations: GS; Gleason score, LN; lymph node, P; prostate | | | | | |
