# Supplementary material for: Prognostic Impact of Different Gleason Patterns on Biopsy Within Grade Group 4 Prostate Cancer
Source: Ann Surg Oncol. 2021 Jun 11;28(13):9179–87. doi: 10.1245/s10434-021-10257-x (PMC8591010; doi:10.1245/s10434-021-10257-x)
Supplement: Supplementary file 2 — Supplementary file2 (DOCX 22 kb) [file 10434_2021_10257_MOESM2_ESM.docx]

Supplementary Table 2

Patient demographics at each institution

|  | ALL | Mayo Clinic | Hamburg-Eppendorf | Weill Cornell | Texas Southwestern |
| --- | --- | --- | --- | --- | --- |
| Number | 1791 | 404 | 1156 | 74 | 157 |
| Median Pre PSA (IQR) | 7.8  (5.3-12.3) | 7.2  (5.0-10.8) | 8.2  (5.7-13.7) | 8.0  (6.0-10.8) | 6.0  (4.0-9.0) |
| Biopsy GS |  |  |  |  |  |
| 3＋5 | 190 (10.6%) | 46 (11.4%) | 121 (10.5%) | 13 (17.6%) | 10(6.4%) |
| 4＋4 | 1557 (86.9%) | 353 (87.4%) | 1001 (86.6%) | 58 (78.4%) | 145 (92.4%) |
| 5＋3 | 44 (2.5%) | 5 (1.2%) | 34 (2.9%) | 3 (4.1%) | 2 (1.3%) |
| cT stage |  |  |  |  |  |
| cT1 | 1010 (56.4%) | 176 (43.6%) | 691 (59.8%) | 42 (56.8%) | 101 (64.3%) |
| cT2 | 700 (39.1%) | 197 (48.8%) | 417 (36.1%) | 31 (41.9%) | 55 (35.0%) |
| ≧cT3 | 44 (2.5%) | 18 (4.5%) | 24 (2.1%) | 1 (1.4%) | 1 (0.6%) |
| missing | 37 (2.1%) | 13 (3.2%) | 24 (2.1%) | 0 | 0 |
| RP GS |  |  |  |  |  |
| GG1 | 18 (1.0%) | 6 (1.5%) | 6 (0.5%) | 0 (0.0%) | 6 (3.8%) |
| GG2 | 489 (27.3%) | 79 (19.6%) | 376 (32.5%) | 8 (10.8%) | 26 (16.6%) |
| GG3 | 711 (39.7%) | 111 (27.5%) | 517 (44.7%) | 26 (35.1%) | 57 (36.3%) |
| GG4 | 244 (30.8%) | 121 (30.0%) | 60 (5.2%) | 31 (41.9%) | 32 (20.4%) |
| GG5 | 322 (18.0%) | 86 (21.3%) | 191 (16.5%) | 9 (12.2%) | 36 (22.9%) |
| missing | 7 (0.4%) | 1 (0.2%) | 6 (0.5%) | 0 (0.0%) | 0 (0.0%) |
| pT stage |  |  |  |  |  |
| ≦pT2 | 783 (43.7% | 221 (54.7%) | 448 (38.8%) | 36 (48.6%) | 78 (49.7%) |
| ≧pT3 | 1002 (55.9%) | 182 (45.0%) | 703 (60.8%) | 38 (51.4%) | 79 (51.3%) |
| missing | 6 (0.3%) | 1 (0.2%) | 5 (0.4%) | 0 | 0 |
| pN stage |  |  |  |  |  |
| N1 | 330 (18.4%) | 56 (13.9%) | 258 (22.3%) | 3 (4.1%) | 13 (8.3%) |
| PSM | 447 (25.0%) | 104 (25.7%) | 309 (26.7%) | 14 (18.9%) | 20 (12.7%) |
| Adj ADT | 113 (6.3%) | 59 (14.6%) | 54 (4.7%) | 0 (0.0%) | 0 (0.0%) |
| Adj RT | 122 (6.8%) | 17 (4.2%) | 105 (9.1%) | 0 (0.0%) | 0 (0.0%) |
| **Abbreviations: Adj; adjuvant, ADT; androgen deprivation therapy, c; clinical, GS; Gleason score, M; months, Neo; neoadjuvant, p; pathological Pre; preoperative, PSM; positive surgical margin, RP; radical prostatectomy, RT; radiation therapy** | | | | | |
