# Supplementary material for: Prognostic Impact of Different Gleason Patterns on Biopsy Within Grade Group 4 Prostate Cancer
Source: Ann Surg Oncol. 2021 Jun 11;28(13):9179–87. doi: 10.1245/s10434-021-10257-x (PMC8591010; doi:10.1245/s10434-021-10257-x)
Supplement: Supplementary file 3 — Supplementary file3 (DOCX 17 kb) [file 10434_2021_10257_MOESM3_ESM.docx]

Supplementary Table 3

Studies examining the prognostic value of the different GS patterns in grade group 4 prostate cancer treated with RP

| Author | Year | N (GS8) | N (GS3+5/GS5+3) | N(GS4+4) | GS specimens | Treatment | Outcomes |
| --- | --- | --- | --- | --- | --- | --- | --- |
| Gandaglia | 2017 | 1089 | 295 (27.1%)/143 (13.1%) | 651 (59.8%) | RP | RP | BCR, CR |
| Ganseler | 2017 | 5474 | 425 (7.8%)/103 (1.9%) | 4946 (90.4%) | Biopsy | RP | GS downgrading |
| Harding-Jackson | 2016 | 179 | 58 (32.4%)/0 (0%) | 121 (67.6%) | Biopsy | Including RP | ACM |
| Lu | 2017 | 740 | 55 (7.4%)/21 (2.8%) | 664 (89.7%) | Biopsy | Including RP | ACM,CSM |
| Mahal | 2016 | 25063 | 2668 (10.6%)/892 (3.6%) | 21503 (85.8%) | RP/biopsy | Including RP  (Subgroup: RP only) | CSM |
| Rusthoven | 2014 | 1073 | 167 (15.6%) | 906 (84.4%) | Biopsy | Including RP | ACM,CSM |
| Van den Bergh | 2016 | 318 | 50 (15.7%)/16 (5.0%) | 252 (79.2%) | RP/biopsy | RP | BCR |
| Mori | 2020 | 787 | 189 (24.0%)/98 (12.5%) | 500 (63.5%) | RP | RP | ACM, CSM, BCR, EPE, NOCD, PSM, Lymph meta |
| Current study | 2021 | 1791 | 190 (9.9%)/44 (2.5%) | 1557 (86.9%) | Biopsy | RP | ACM, CSM, BCR, GS upgrading. EPE, NOCD, PSM, Lymph meta |
| **Abbreviations: ACM: all-cause mortality, ADT:androgen deprivation therapy, BCR: biochemical recurrence, CR: clinical recurrence, CSM: cancer-specific mortality, EPE: extraprostatic extension, GS: Gleason score, Lymph meta: lymph node metastasis, NOCD: non-organ-confined disease, PSM: positive surgical margin, RP: radical prostatectomy** | | | | | | | |
